# QbD-Based Development and Evaluation of Pazopanib Hydrochloride Extrudates Prepared by Hot-Melt Extrusion Technique: In Vitro and In Vivo Evaluation

**DOI:** 10.3390/pharmaceutics16060764

**Published:** 2024-06-04

**Authors:** Amit Gupta, Rashmi Dahima, Sunil K. Panda, Annie Gupta, Gaurav Deep Singh, Tanveer A. Wani, Afzal Hussain, Devashish Rathore

**Affiliations:** 1 School of Pharmacy, Devi Ahilya Vishwavidyalaya, Takshashila Campus, Ring Road, Indore 452001, India; drrashmidahima@gmail.com (R.D.); devashish28sopdavv@gmail.com (D.R.); 2 Research & Development, GM Pharmaceutical Inc., 0114 Tbilisi, Georgia; sunil.kumar@gmpgeo.com; 3 Amity Institute of Pharmacy, Amity University, Sector 125, Noida 201303, India; 4 Department of Chemistry, Radha Govind University, Ramgarh 829122, India; 5 Department of Pharmaceutical Chemistry, College of Pharmacy, King Saud University, Riyadh 11451, Saudi Arabia; twani@ksu.edu.sa; 6 Department of Pharmacognosy, College of Pharmacy, King Saud University, Riyadh 11451, Saudi Arabia; afihussain@ksu.edu.sa

**Keywords:** quality by design (QbD), design of experiments (DoE), extrudates, disintegrant, hot-melt extrusion, HME polymer, solid dispersion

## Abstract

Background: Pazopanib hydrochloride (PZB) is a protein kinase inhibitor approved by the United States Food and Drug Administration and European agencies for the treatment of renal cell carcinoma and other renal malignancies. However, it exhibits poor aqueous solubility and inconsistent oral drug absorption. In this regard, the current research work entails the development and evaluation of the extrudates of pazopanib hydrochloride by the hot-melt extrusion (HME) technique for solubility enhancement and augmenting oral bioavailability. Results: Solid dispersion of the drug was prepared using polymers such as Kollidon VA64, hydroxypropylmethylcellulose (HPMC), Eudragit EPO, and Affinisol 15LV in a 1:2 ratio by the HME process through a lab-scale 18 mm extruder. Systematic optimization of the formulation variables was carried out with the help of custom screening design (JMP Software by SAS, Version 14.0) to study the impact of polymer type and plasticizer level on the quality of extrudate processability by measuring the torque value, appearance, and disintegration time as the responses. The polymer blends containing Kollidon VA64 and Affinisol 15LV resulted in respective clear transparent extrudates, while Eudragit EPO and HPMC extrudates were found to be opaque white and brownish, respectively. Furthermore, evaluation of the impact of process parameters such as screw rpm and barrel temperature was measured using a definitive screening design on the extrude appearance, torque, disintegration time, and dissolution profile. Based on the statistical outcomes, it can be concluded that barrel temperature has a significant impact on torque, disintegration time, and dissolution at 30 min, while screw speed has an insignificant impact on the response variables. Affinisol extrudates showed less moisture uptake and faster dissolution in comparison to Kollidon VA64 extrudates. Affinisol extrudates were evaluated for polymorphic stability up to a 3-month accelerated condition and found no recrystallization. PZB–Extrudates using the Affinisol polymer (Test formulation A) revealed significantly higher bioavailability (AUC) in comparison to the free Pazopanib drug and marketed formulation.

## 1. Introduction

Oral bioavailability and efficacy are significantly impacted by drug solubility; around 70% of newly discovered medications have poor bioavailability [1]. This problem appears to be more common with anti-cancer medications. About 90% of novel chemical entities for formulation development in pharmaceutical industries pipelines have the drawback of having low aqueous solubility [2]. Formulation experts have thus found it difficult to construct a dosage form for such compounds. Solid dispersion is a promising formulation strategy to improve the oral bioavailability of poorly aqueous-soluble drugs by increasing gastrointestinal solubility and drug absorption into the systemic circulation [3]. Solid dispersion (SD) is a monophasic system in which the active pharmaceutical ingredient (API) is dispersed in a carrier. It has a lower melting point in comparison to its component [4]. The preparation of a solid dispersion of a poorly soluble drug leads to changes in the physical state of the drug on the molecular level. The physical state is dependent on various factors such as physiochemical properties of the carrier, drug compound (API), drug–carrier interactions, and method of preparations [5,6]. Solid dispersions can be prepared by different approaches such as the melting method, hot-melt extrusion (HME) process, melt agglomeration, spray drying, fusion, solvent evaporation, supercritical anti-solvent (SAS), electrostatic spinning, etc. [7,8,9]. However, the HME technique is extremely suitable for preparing solid dispersion in comparison to other solid dispersion preparation methods. Since it is a simple, high-throughput, robust process with a limited number of processing steps and does not require any solvent or water. Fluffy and poor-flow material can be compacted easily with minimal dust generation by this technique [6,10]. HME is a solvent-free continuous process with proven industrial applicability for the enhancement of solubility of poorly soluble compounds [11]. 

In the hot-melt extrusion (HME) technique, a mixture of a drug substance and polymeric carrier is passed through the heated barrel and melted homogenous mass is discharged in the form of extrudates. The amalgamation of the mixture with force by spinning the rods leads to the disaggregation of the drug substance within the polymeric carrier, which results in the formation of a homogenous drug dispersion [12,13]. It requires one or more melted polymeric carriers and other additives like plasticizers, surfactants, and other pH modifiers. It is a well-known process to produce a polymer product of uniform shape and density [14]. It has diverse applications such as improving the solubility of drugs, taste masking of bitter drugs, sustained drug release, and preparing abuse-deterrent formulations [15,16]. Hence, the HME technique has promising potential for the solubility and bioavailability enhancement of drugs, thus reducing dose-related toxicological side effects [17]. 

Polymers with high solubilization potential are particularly useful in the HME process; hence, large quantities of drug substance can be solubilized [18]. Some characteristics of HME polymers such as hydrogen bonding donors or acceptors, presence of an amide group, lipophilicity, etc., are basic prerequisites that are responsible for their solubilization capacity [19]. The selection of a suitable polymeric carrier for the HME process is an important aspect that should be evaluated to ensure the robust development of drug extrudates. The polymer must have the desired physicochemical properties such as thermoplastic behavior, thermal stability, and no decomposition during the extrusion process [20]. The real indicator of thermo-plasticity is glass transition temperature (Tg), and the polymers with high Tg values indicate that higher processing temperatures are needed to prepare solid dispersion by the HME technique [21]. Thus, in order to reduce the Tg value of the physical mixture, a plasticizer such as Poloxamer 188, polyethylene glycol (PEG), Triethylcitrate (TEC), etc., may be used [22,23]. 

The polymers commonly employed to form solid dispersions by the HME technique have high binding and gelling properties; hence, their presence at a high level in the solid dispersion can result in a very long disintegration time of drug extrudates, tablets, or finished dosage form. These polymers are polyvinylpyrrolidone (PVP), Kollidon VA64, hydroxypropylmethylcellulose (HPMC), hydroxy propyl cellulose (HPC), etc. [24,25,26].

Currently, protein kinase inhibitors (PKIs) are the frontline drugs for the treatment of both locally advanced and metastatic cases of cancer [27,28]. Most PKIs, including Pazopanib HCl (PZB), have low water solubility and low (BCS Class IV) or high permeability (BCS Class II compound). Pazopanib HCl is a weak base with pH-dependent solubility that solubilizes at a low pH and is practically insoluble at a neutral pH (0.1–10 mg/mL) [29,30]. Therefore, it is an essential need to develop and enhance the solubility and drug release characteristics of Pazopanib HCl (PZB) to improve the solubility and bioavailability.

The aim of the present study, therefore, was to prepare the solid dispersion of PZB using a suitable polymer blend for improving the aqueous solubility of the drug and enhancing systemic drug absorption. The critical process parameters during extrudate preparation and its impact on processibility, appearance, disintegration time, and dissolution were also studied. As mentioned earlier, the extrudates were prepared employing polymer blends such as Kollidon VA64, Affinisol, Eudragit EPO, and HPMC, and rationally optimized using experimental designs in the current study for understanding the impact of formulation and process variables on quality, dissolution performance, and pharmacokinetic drug absorption. Pazopanib HCl drug extrudates consist of 31.25% w/w Pazopanib HCl (PZB) drug substance and 62.5% *w*/*w* polymer loading and are at a fixed poloxamer 188 level (10% *w*/*w* of polymer). It is hypothesized that HME polymer physicochemical properties will have a major impact on the quality of the drug extrudates, since the polymer is a major component. The outcomes from this study may provide a way forward for other researchers in developing HME tablets, capsules, or other solid dosage forms with better processability and desired attributes, such as faster disintegration time and dissolution, for the chosen HME polymers.

## 2. Materials and Methods

### 2.1. Materials

Pazopanib Hydrochloride (PZB) [CAS Number 635702-64-6, Molecular weight—473.99, Water by KF—0.06% *w*/*w* and Purity—100.2% *w*/*w* on anhydrous basis] was gifted from Sun Pharmaceutical Industries Limited (Gurgaon, India). Hydroxypropylmethylcellulose (Hypromellose 5cps) [CAS Number—9004-65-3, Methoxy content—28.0 to 30.0%, Hydroxypropyl content—7.0 to 12.0%] was obtained from Dow chemicals as a gift sample. The methacrylate polymer Eudragit EPO [CAS Number—24938-16-7, Molecular weight—47,000 g/mol] was obtained from Evonik Industries (Mumbai, India). The cellulosic polymer Affinisol HPMC HME 15LV [Catalog—HH127947, CAS Number—9004-65-3, Tg—115 °C] was donated by Dupont (Nutrition and Biosciences, New York, NY, USA). Kollidon VA—64 vinylpyrrolidone polymer [CAS Number—25086-89-9] and Poloxamer P188 [CAS Number—9003-11-6, Average molecular weight—7680 to 9510] as plasticizer were obtained from BASF Corporation (Mumbai, India). All other chemicals and solvents used in this study were of analytical grade.

### 2.2. Methods

#### 2.2.1. Prototype Formula Composition

A prototype formula was designed for manufacturing of Pazopanib HCl solid dispersion by using Hot-melt extrusion technique. Commonly used polymers and a plasticizer (poloxamer 188) [BASF Pharma, Navi Mumbai, India] were employed for preparation of solid dispersion. Drug-to-polymer and plasticizer-to-polymer ratios were kept constant at 1:2 and 1:10, respectively. Formula compositions are mentioned below in Table 1. 

#### 2.2.2. Preparation of Drug Extrudates by Hot-Melt Extrusion

Different batches of extrudates were prepared by HME using co-rotating twin-screw extruder (Model: Pharma 11, Thermoscientific, Dreieich, Germany) fitted with various screws containing kneading elements at 30° and 60° orientation. For this study, the feed rate of the pre-mix material and the screw speed were kept constant for all experimental trials at 2 g/min and 100 rpm, respectively. Barrel temperatures dependent on the thermal properties of the formulation components were kept constant for all experimental trials. The thermally stable polymers taken for the preparation of extrudates were Kollidon VA 64, Eudragit EPO, HPMC, and Affinisol 15LV, which are stable up to temperature >200 °C. Different polymers were mixed with the drug and the plasticizer, and the prepared pre-mix was passed through the twin-screw extruder [31]. The torque was measured during HME process, since it is a key indication of processibility, and was recorded. The extrudates were kept at room temperature prior to milling operation. A Quadro co-mill (Model:197 Overdriven Comil, Idex Corporation, Rohnert Park, CA, USA) was operated at low speed (1100 rpm) with forward knives and a 40 G (1.00 mm) screen to mill the extrudates. The 16 # BSS (1003 µm) to 60# BSS (250 µm) powder fraction was collected and further used for in-process analysis.

#### 2.2.3. Systematic Optimization of Formulation Variables

To understand the impact of formulation variables such as polymer type and plasticizer concentration on extrudate quality, a systematic experimental approach, i.e., design of experiment (DoE) was employed. The experimental design consisted of two input variables, one categorical (polymer type) and one continuous (plasticizer levels) factor. The input variables with their study levels are described in Table 2. Since the variables are categorical and continuous, a custom design was chosen. Systematic experiments for the suggested design were planned using JMP software (version 14.0 by SAS) for modeling and data analysis. The response (dependent) variables were extrudate appearance, machine torque, and disintegration time. 

#### 2.2.4. Optimization of Process Parameters/Variables

The impact of critical process parameters such as screw rpm and barrel temperature on torque, appearance, DT, and dissolution were evaluated by systematic optimization. Kollidon VA64 and Affinisol 15LV were chosen for further studies based on formulation DoE outcomes. A systemic DoE design consisted of three factors—one categorical (polymer type) and two continuous factors (screw speed and barrel temperature). The factors and responses are described above in the table (Table 2: DoE Matrix for Formulation and Process Variables). Pazopanib hydrochloride (PZB) drug was mixed with optimized HME polymer (Kollidon VA64 and Affinisol 15LV) in a ratio of 1 to 2 and poloxamer 188 (10% *w*/*w* of polymer weight), which resulted in a drug concentration of 31.25%. The trials were carried out on a twin-screw extruder equipped with kneading elements as before.

### 2.3. Characterization Studies and Stability Study of Final Prototype

#### 2.3.1. Density and Particle Size Distribution of Milled Extrudates

The bulking properties of the milled extrudates depend on the polymer type and preparation method. Bulk density/Tapped density (BD/TD) of the material was carried out according to method I of USP chapter <616> “Bulk density and Tapped density of powders”. Particle size distribution (PSD) analysis of milled extrudates prepared using different HME polymers was performed by sieving method. The sieve analysis was performed using an electromagnetic sieve shaker instrument (Model AS200, Retsch, Haan, Germany) in which sieves vibrate by throwing motion with angular momentum. Approximately 5 g of sample was taken and sifted through 16# BSS (1003 µm) and 60#BSS (250 µm) sieves, with the pan and sieve shaker set at 10% amplitude on continuous mode for 5 min. 

#### 2.3.2. Dissolution Testing under Sink Conditions

Dissolution test was performed on PZB-drug, a physical mixture of optimized polymer, i.e., Kollidon VA 64 and Affinisol 15 LV polymer and its extrudates, using USP Type II (paddle) apparatus [Electrolab, Navi Mumbai, India]. The dissolution media were chosen as recommended in the United States Food and Drug Administration (USFDA) dissolution database. The dissolution was conducted in 50 mM, pH 4.5 sodium acetate buffer with 0.75% sodium lauryl sulfate (SLS) in 900 mL at 75 rpm paddle speed, and temperature was set at 37  ±  0.5 °C. Prior to testing, media were degassed and pre-heated to 37 °C in each transparent dissolution glass vessel. Approximately 200 mg equivalent of the PZB-drug samples (n = 6) was weighed and transferred into the dissolution vessel. Sampling was carried out at different time intervals (15 min, 30 min, 45 min, 60 min) and about 5 mL of the sample was pulled out using syringe and filtered through a 0.45 μm PVDF membrane filter. A fresh 5 mL preheated buffer media was transferred immediately after sampling. Filtered samples were further analyzed by using UV spectrophotometer (Shimadzu, Tokyo, Japan) at 270 nm wavelength [32]. 

#### 2.3.3. Moisture Uptake Study/Hygroscopicity Study

Low moisture sorption is crucial for solid dispersion extrudates prepared by the HME technique [23]. The presence of water may likely plasticize the drug extrudates, resulting in a reduction in the glass transition temperature. The hygroscopicity of the PZB-drug, polymers, and drug extrudates was calculated gravimetrically. Accurately 1 g of samples was kept and stored in opened glass vials under accelerated stability conditions (40 ± 2 °C and 75 ± 5% relative humidity) and long-term storage conditions (25 ± 2 °C and 60 ± 5% relative humidity) for 7 days. The samples were removed from the stability chamber for hygroscopicity analysis and the percent weight gain was calculated.

#### 2.3.4. Hot-Stage Microscopy (HSM)

Hot-stage microscopy (HSM) was conducted using a hot stage under a polarizing optical microscope (Leica Microsystems GmbH, Wetzlar, Germany) [33]. The attached camera shows the live image of the sample during the heat treatment and material phase transition during the process was observed. 

The prepared samples were placed on open glass slides and kept on the hot-stage plate and heated from 30 to 300 °C at 10 °C/min and the changes in physical state were visually observed. The images were captured whenever a transition occurred and the temperature was noted down accordingly. The images were captured using Canon camera under polarizing light for further analysis. 

#### 2.3.5. Powder X-ray Diffraction (PXRD)

PXRD was carried out to assess the physical state of Pazopanib HCl (PZB-drug), a polymorphic form of initial Affinisol extrudates, and a 3-month accelerated stability sample. PXRD was performed using a D/Max-2400 X-ray Fluorescence Spectrometer (Rigaku, Osaka, Japan) with a Cu Ka line as the source of radiation, and standard runs using a voltage of 56 kV, a current of 182 mA, and a scanning rate of 2°/min over a 2-theta from 3° to 45° were carried out.

#### 2.3.6. Stability Studies

To assess the physical stability of the final prototype formulation (Affinisol PZB-extrudates) regarding the recrystallization of Pazopanib (PZB) drug, milled extrudates (Affinisol 15 LV extrudates) in LDPE-capped glass vials were stored at accelerated and long-term stability conditions for up to 3 months. The samples were analyzed for description, assay, and dissolution. 

#### 2.3.7. Pharmacokinetics Studies

A bioavailability study was carried out to assess the drug absorption and pharmacokinetic parameters of different prepared formulations [34]. A suitable animal was selected, and in vivo studies were performed in accordance with the ethical guidelines for animal care and use committee. 

New Zealand white rabbits (Species and strain: *Lupus caniculus*) (5–6 months older, 2.0 ± 0.2 kg, 34–50 cm without tail) were utilized in this study. Animals were maintained at standard environmental conditions of temperature 24 ± 2 °C, humidity (55 ± 5%), under 12:12 h light (08:00–20:00 h)/dark cycle. Animals had free access to rodent chow and tap water ad libitum. Approval for animal studies was gained by the Institutional Animal Ethics Committee (Protocol no. IAEC; 1888/PO/Re/S/16/ CPCSEA/2022/01), constituted for the purpose of control and supervision of experimental animals and agreed with the globally acknowledged Principles for Laboratory Animal Use and Care as indicated by the National Institutes of Health Guide (NIH Publication No. 85–23, revised 1985). Animals were naive to drug treatments and experimentation, at the beginning of all studies. All tests were conducted at BM College of Pharmaceutical Education and Research (BMCPER), Indore, M.P. between 09:00 and 18:00 h.

Animals (n = 3) were settled into three different groups. First group (control) received Pazopanib drug (control), second group received the marketed pazopanib hydrochloride immediate release tablet (Votrient Tablets, 200 mg), while third group received drug formulations (equivalent to 200 mg of Pazopanib) prepared using HME technique, respectively. Dosage forms were administered orally via gastric intubation. Animals were fasted for 12 h with free access to water prior to drug administration. Rabbits were held in rabbit restrainers during blood sampling. Blood samples were collected in heparinized tubes from ear veins at predetermined time points (0, 0.5, 1, 2, 3, 4, 6, 8, 12, and 24 h) after oral administration. Plasma samples were obtained following centrifugation of blood at 3200× *g* for 5 min at 2–8 °C and kept frozen at −70 °C until analysis [35].

#### 2.3.8. Statistical Analysis

To compare different batches during optimization studies of formulation and process variables, statistical analysis was performed utilizing one-way analysis of variance (ANOVA). A statistically significant difference was considered when *p* value < 0.05.

## 3. Result and Discussion

In this study, Pazopanib HCl, a weak basic drug molecule with pH-dependent solubility belonging to the BCS class II category, was used. It has poor aqueous solubility, with moderate hydrophobicity (log P of 3.55) and 2.5 pKa [36]. 

The thermal characteristics of the chosen drug Pazopanib HCl (PZB) have been discussed in the research article titled ‘Thermal Study of Pazopanib hydrochloride”, which states that the Pazopanib HCl (PZB) drug substance has first an endothermic peak between 48 °C and 125 °C and then an exothermic peak at 238 °C and a sharp endothermic peak above 280 °C. The primary endothermic peak corresponds to the loss of water from the drug substance, and the subsequent exothermic peak is due to the loss of HCl. The last sharp endothermic peaks may be due to structural degradation [37]. Therefore, no melting point peak was observed, which was also not observed during melting point determinations. 

The particle size distribution of Pazopanib drug (PZB) was found to be D_90_; 65.2 μm, D_50_; 29 μm, and D_10_; 7 µm. The bulk density (BD) and tap density (TD) were found to be 0.25 g/mL and 0.42 g/mL, respectively. The resultant Carr index (CI) value was calculated and found to be 40.4, suggesting the fluffy nature and poor flow characteristics of the Pazopanib HCl (PZB) molecule.

### 3.1. Outcome for Optimization of Formulation Variables

The table below summarizes the studied outcome for the effect of polymer type and plasticizer level on the appearance, torque, and DT of the manufactured extrudates. 

#### 3.1.1. Effect of Polymer Type on Appearance of Extrudates and Processability (Torque)

To investigate the impact of the formulation components on the extrudates’ quality, the polymer type and plasticizer concentrations were varied. The final aim of the current study was to choose the suitable polymer, which led to better miscibility with the drug, and prepare a solid dispersion with easy HME processibility and minimal loss of drug content. Maddineni et al. also investigated the impact of formulation and process variables on the characteristics of Nifedipine extrudates; prepared by using Kollidon VA 64 polymer [38].

The decomposition temperature of various utilized polymers has been reported, such as 230 °C for Kollidon VA64, 200 °C for Eudragit EPO, and 200–250 °C for HPMC [39]. These findings suggest that the processing temperature should be kept below 180 °C and that other parameters, such as screw speed and screw design, be tuned in accordance with the extrusion results. The HME extrusion process of the Pazopanib drug (PZB) substance with each polymer (Kollidon VA64, Eudragit EPO, Affinisol 15LV, and HPMC) was conducted at extrusion temperature 160–180 °C in the extruder. For batches containing Affinisol 15LV and Kollidon VA64, light yellow transparent extrudates were obtained, while for the Eudragit EPO- and HPMC-containing batches, the extrudates were less transparent with opaque white and brownish color being observed, respectively. Hence, this indicated a good mixing of PZB-drug with Affinisol 15LV and Kollidon VA64, respectively. 

The results were in accord with the findings of authors Huang et al. for carbamazepine drug extrudates that were prepared using various hot-melt extrusion polymers; the carbamazepine extrudates were found to be clear and transparent in appearance with the studied polymer, i.e., Soluplus, Kollidon VA 64, and different grades of Affinisol, except for the Eudragit EPO formulation [40]. 

The selected polymers, except HPMC, have a degradation temperature >220 °C, while the HPMC degradation temperature is around 190 °C. Since the processing temperature was not more than 180 °C, no significant change in the extrudates’ color was observed; only some changes in polymer structure might have occurred, which resulted in brownish color extrudates.

The appearance of the extrudates was clear and transparent with Affinisol and Kollidon VA64, which might be due to low-melt viscosity, while there was a white opaque color with Eudragit EPO. The extrudates were browning in color with cellulosic polymer HPMC, which might be due to the charring of the polymer. LaFountaine et al. prepared HPMC-based extrudates and found a similar brown color appearance [41], which is concomitant with the present experimental observations.

During the extrusion process, the torque values were observed to be 6.4 and 5.6 Nm respectively for the pre-mix containing the polymers Affinisol 15LV and Kollidon VA64 in the absence of a plasticizer, while no significant differences in torque values were observed with the use of a plasticizer. For both polymers, a moderate torque value suggests favorable processing conditions. Therefore, the HME extrusion processibility was good, which could be due to the moderate melt viscosities of Affinisol 15LV and Kollidon VA64. Moreover, the torque values were observed to be comparatively higher with both the HPMC and Eudragit EPO polymers, which might be attributed to the higher melt viscosities of these polymers. The use of HPMC and Eudragit EPO as polymers required a plasticizer to reduce the viscosity of the polymer melt; otherwise, the torque value was likely to be exceeded. Therefore, the plasticizer level was also evaluated (0% *w*/*w*—without plasticizer and 10% *w*/*w*). Poloxamer 188 was selected as a plasticizer as it was shown to be the most suitable to reduce the melt viscosity of polymer and decrease the torque or motor load of the extruder machine. The cleaning of the extruder machine was quite difficult after the extrusion process of the Eudragit EPO and HPMC batches compared to the Affinisol 15LV and Kollidon VA64 batches. Benett et al. incorporated triethyl citrate (TEC) and acetyl tributyl citrate (ATBC) for the preparation of griseofulvin extrudates by the HME process, and the results revealed that the plasticizers reduced the melt viscosities during the extrusion process. Therefore, by increasing the free volume among polymer chains, plasticizers can be used as processing aids to lower component glass transition temperatures and melt viscosities [42]. Moreover, plasticizers can have a detrimental effect on the stability of solid dispersions even when they help in fusion processing. There is a greater chance of crystallization as molecular mobility rises [43].

The milling process was carried out using a Quadro Comill fitted with a 40 G screen at 700 rpm (slow speed). At 500 rpm, the extrudates prepared using HPMC were not milled; however, extrudates prepared using other polymers were milled at a slow speed. 

#### 3.1.2. Effect of Polymer Type and Plasticizer on Disintegration Time of Extrudates

Various trials were conducted using different polymers with (trial no. HME-1 to HME-4) and without plasticizers (trial no. HME-5 to HME-8), and their impact on the disintegration time (dependent variable) was evaluated. The disintegration time with extrudates containing HPMC, Eudragit EPO, Affinisol 15LV, and Kollidon VA64 was 15 min 32 s, 23 min 6 s, 11 min 45 s, and 18 min 11 s, respectively, in the presence of 10% *w*/*w* of poloxamer 188 as plasticizer (refer to Table 3 for trials no. HME-1 to HME-4). Poloxamer is a triblock copolymer and can be used to enhance the solubility and dissolution rate of poorly soluble drugs [44]. Additionally, Karekar et al. discovered that Etoricoxib solid dispersion which was prepared by using poloxamer 188 shown superior solubility and dissolution rate. Poloxamer is frequently employed in ASDs as a surfactant [45].

The results indicate that polymer type has a strong influence on the disintegration time of drug extrudates. 

Furthermore, the disintegration time was slightly on the higher side when drug extrudates were prepared without using a plasticizer, and DT was observed 19 min 18 s, 27 min 16 s, 13 min 46 s, and 22 min 54 s in extrudates containing HPMC, Eudragit EPO, Affinisol 15LV, and Kollidon VA64, respectively (refer to Table 3 for trials no. HME-5 to HME-8). 

Based on these results, it can be concluded that the longest disintegration time of extrudates in water media was found with Eudragit EPO polymer, followed by Kollidon VA64 and HPMC, and the shortest DT was achieved with Affinisol 15LV polymer. 

Kollidon VA64 is a water-soluble vinylpyrrolidone-vinyl acetate copolymer, with good binding and compatibility properties. Strojewski et al. discussed that copovidone polymer can be used to enhance both dissolutions as well as in vivo bioavailability [46]. Affinisol HPMC is a modified hypromellose, designed by architectural substitution that expands the thermal processing window and increases the solubility in the organic solvent. It is a water-soluble amorphous cellulosic polymer that may help to enhance the solubility of poorly soluble drugs by forming stable solid dispersions and also inhibiting drug substance crystallization. Affinisol 15LV is a white-to-off-white free-flowing powder with approximately (Tg) and remains thermally stable above 250 °C. The recommended lowest and highest processing temperatures for Affinisol HPMC 15LV polymers are 135 °C and 190 °C, respectively [47]. 

Eudragit EPO is a cationic copolymer of polymethacrylates with low miscibility. It is a white powder with a characteristic amine-like odor. Eudragit EPO is an excellent matrix former and is used for the sustained release of drugs or enteric coatings. Mendes et al. prepared an amorphous solid dispersion of poorly soluble sulfamethoxazole drug to maintain the supersaturation in a biorelevant medium by spray drying and the hot-melt extrusion method. When the Eudragit EPO polymer was employed, the results show that supersaturation was maintained up to 24 h at all the drug–polymer proportions [48].

#### 3.1.3. Statistical Evaluation

##### Influence of Formulation Variables (Formula DoE) on Torque & DT

**Figure 1:**
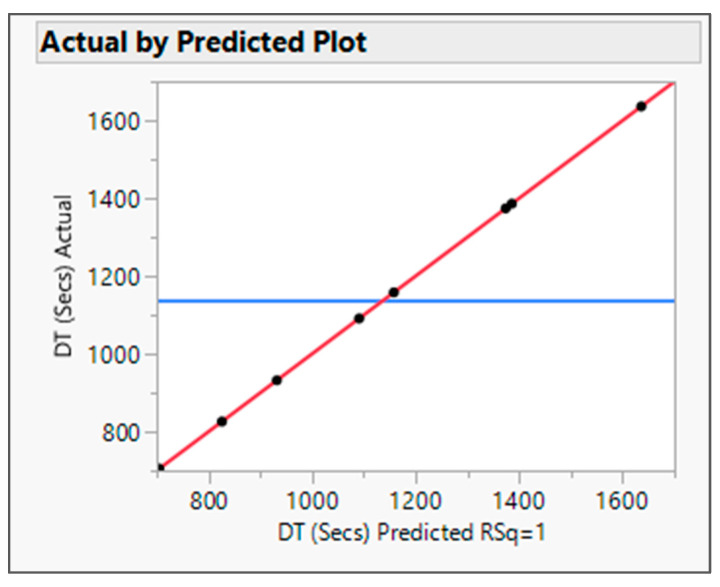
Actual by predicted plot for response DT. Blue Line-for the null hypothesis, Red Line-For Alternative Hypothesis.

**Figure 2:**
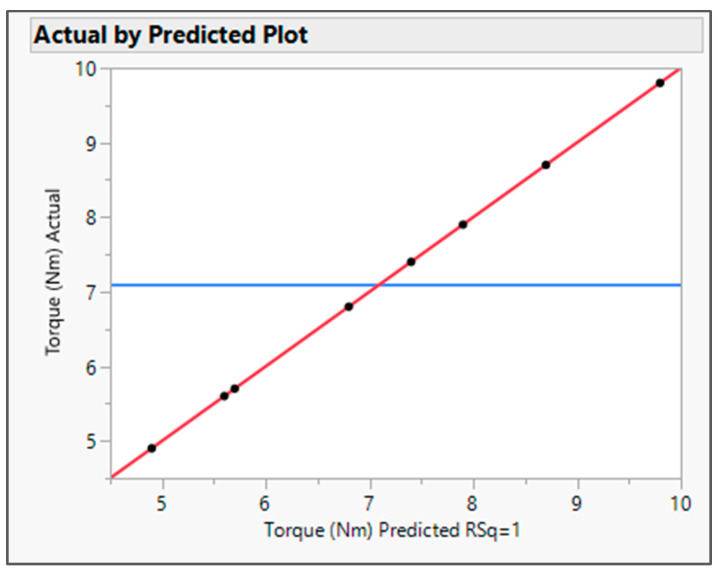
Actual by predicted plot for response Torque. Blue Line-for the null hypothesis, Red Line-For Alternative Hypothesis.

**Figure 3:**
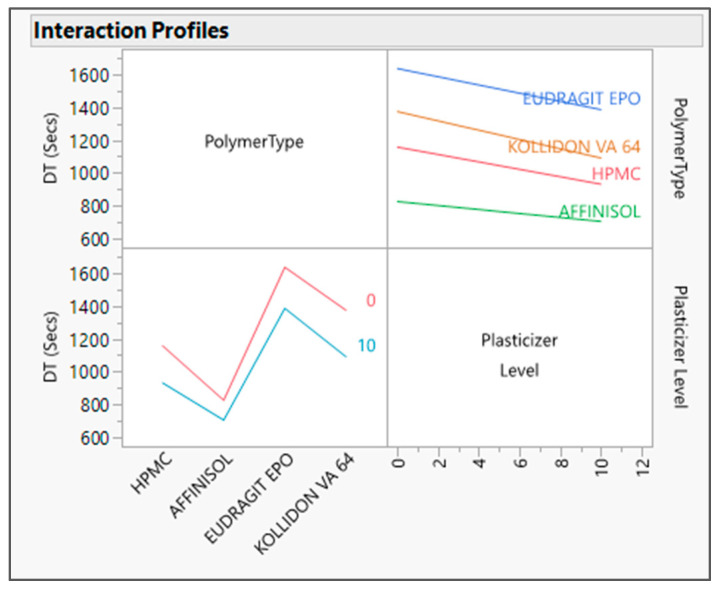
Interaction profiler between input variables and its impact on DT.

**Figure 4:**
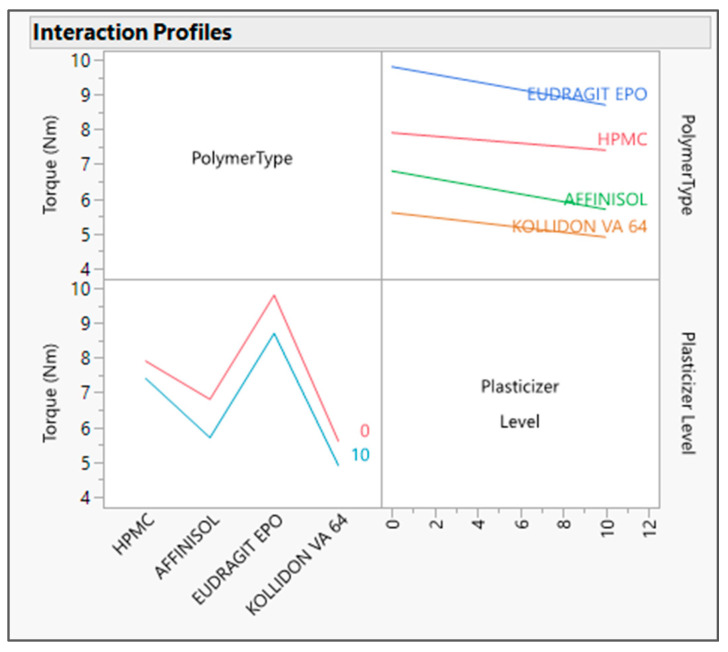
Interaction profiler between input variables and its impact on Torque.

The output variable data were analyzed by fitting the standard least squares model in JMP software. The R Square value for both the output variables was found to be about one in the actual by the predicted plot for DT (Figure 1) and Torque (Figure 2), which defines the strong correlation between actual and predicted values. From the interaction profiler for response DT (Figure 3), it can be concluded that extrudates prepared with the Affinisol polymer had the minimum DT, while extrudates prepared using Eudragit EPO showed the maximum DT. The interaction profiler (Figure 4) showed that the extrudates prepared with the Kollidon VA64 polymer revealed the minimum torque value, while Eudragit EPO extrudates were found with the maximum torque value. Based on these findings, it can be concluded that both Affinisol 15LV and Kollidon VA64 extrudates are promising formulation prototypes for further optimization studies.

**Figure 5:**
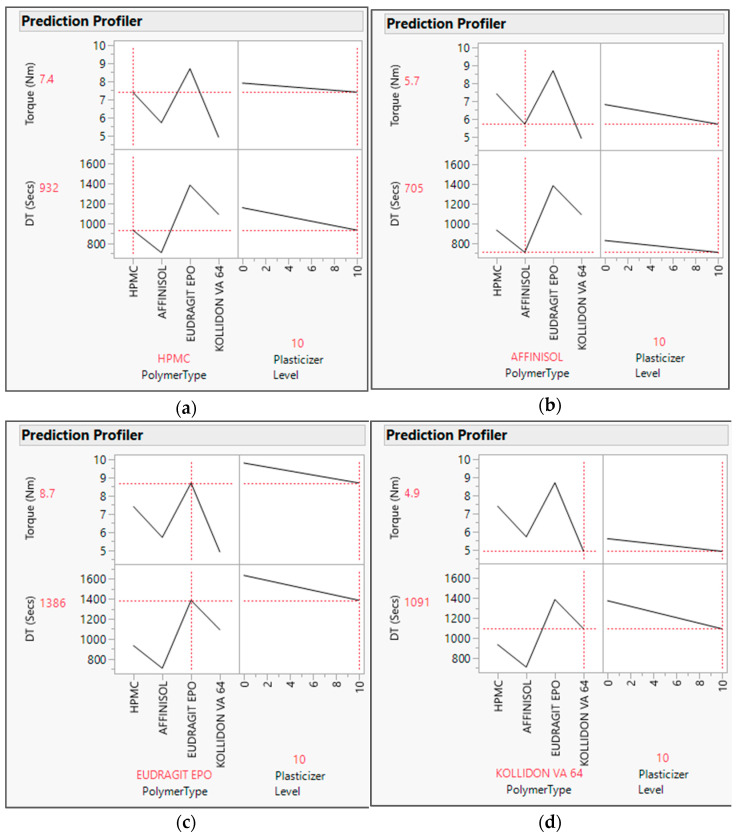
Prediction profilers for output variable with Polymer type and Plasticizer level concentration: (**a**) HPMC + 10% plasticizer, (**b**) Affinisol + 10% plasticizer, (**c**) Eudragit EPO + 10% plasticizer, (**d**) Kollidon VA64 + 10% plasticizer. The red dashed line in the prediction profiler shows the relationship for chosen input variable (on X-axis) and its response/output variable value (on Y-axis). Black solid line depicts the trending for studied input & its output variables.

Prediction profilers are presented below with each studied polymer with a 10% *w*/*w* plasticizer level, i.e., HPMC [Figure 5a], Affinisol [Figure 5b], Eudragit EPO [Figure 5c], and Kollidon VA 64 [Figure 5d]. As per the prediction profiler, the torque and DT values decreased as the plasticizer concentration increased from 0 to 10%. Hence, the plasticizer concentration has impact on both output variables. 

The prediction profilers above also signify that at 10% *w*/*w* concentration of the plasticizer level, the predicted values by model for the response torque were found to be 7.4, 5.7, 8.7, and 4.9 Nm, and for disintegration time was found to be 932, 705, 1386, and 1091 s with HPMC, Affinisol, Eudragit EPO, and Kollidon VA64 polymers, respectively. 

### 3.2. Characterization of Milled Extrudates by Density and Particle Size Distribution

Extrudates can be milled to significantly enhance surface area and, consequently, API diffusion [49,50]. The extrudates were fractionated in order to further study and corroborate this. The bulk density of the material depends on the density and the spatial arrangement of particles [51,52]. Bulk density (BD), tapped density (TD), and particle size distribution (PSD) of milled extrudates were measured and, further, the Hausner ratio and Carr’s index were also calculated. 

**Figure 6:**
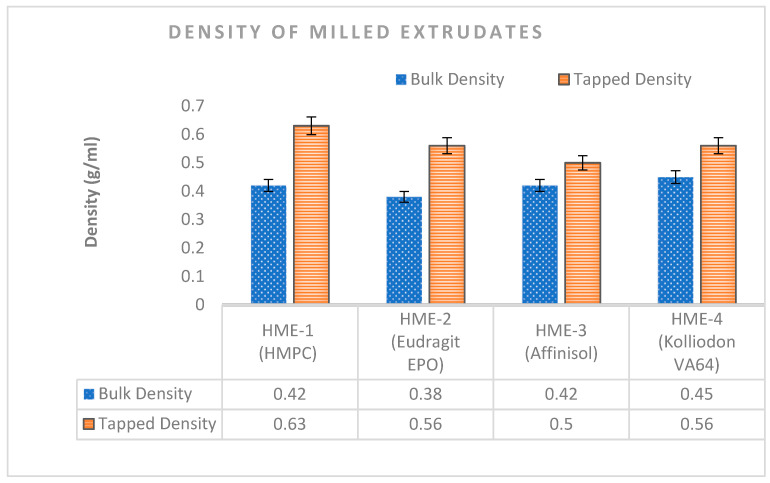
Bulk and tapped density of Pazopanib HCl (PZB)-milled extrudates containing HPMC, Eudragit EPO, Affinisol, and Kollidon VA64.

Different polymeric extrudates have a unique brittle characteristic due to the glassy nature of extrudates [53]. The results of the bulk density and the tapped density of the PZB-milled extrudates with each polymer are summarized in Figure 6. The densities of prepared milled extrudates using HMPC and Affinisol 15 LV were found to be similar, which might be due to the similar chemical characteristics of both polymers. Moreover, the bulk density for milled extrudates with Eudragit EPO and Kollidon VA64 polymers was found to be 0.38 g/mL and 0.45 g/mL, respectively. Based on the density value, it can be concluded that the milled extrudate batches with Affinisol 15 LV and Kollidon VA 64 have fewer inter-particulate interactions since the difference in the bulk and tapped densities values were low, i.e., 0.08 g/mL and 0.11 g/mL, respectively, while the difference in the bulk and tapped densities values was comparatively higher, i.e., 0.21 g/mL and 0.18 g/mL, respectively, in milled extrudates with HPMC and Eudragit EPO polymers.

**Figure 7:**
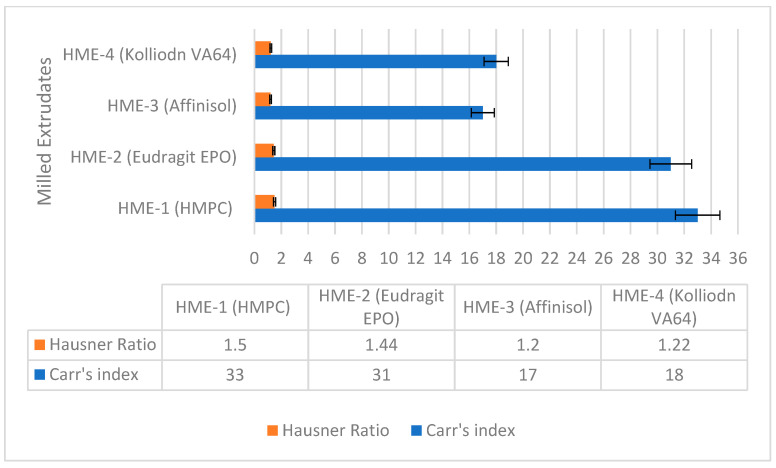
Hausner ratio and Carr’s index of Pazopanib HCl (PZB) containing HPMC, Eudragit EPO, Affinisol, and Kollidon VA64.

As discussed in USP Chapter <616> ‘Bulk density and tapped density of powders’, inter-particulate interactions also interfere with the flowability of material. Furthermore, the Hausner ratio (HR) and Carr’s index (CI) were also calculated for each polymer containing milled extrudates to compare the flow and compressibility characteristics [54] The results of Hausner’s ratio (HR) and Carr’s index (CI) of the milled extrudates are summarized in Figure 7. The HR value and the CI value were in decreasing order for the milled extrudates prepared using HMPC, Eudragit EPO, Kollidon VA64, and Affinisol 15 LV, indicating increased powder flowability. 

**Figure 8:**
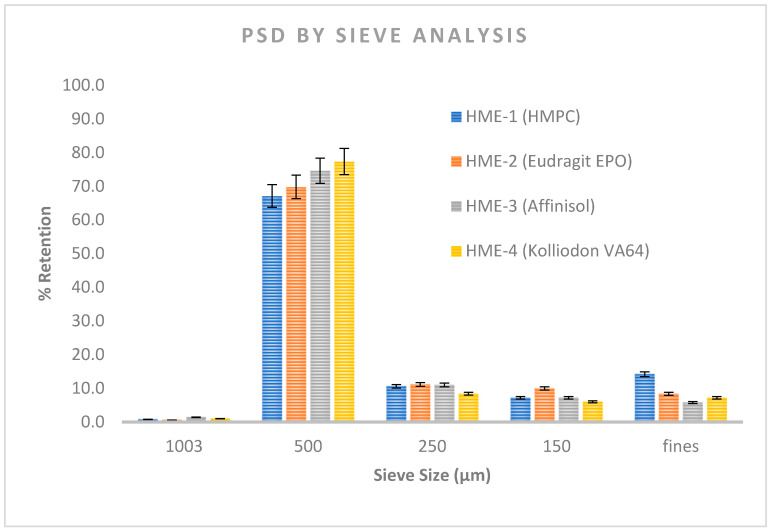
Sieve analysis results of Pazopanib HCl (PZB)-milled extrudates containing HPMC, Eudragit EPO, Affinisol, and Kollidon VA64.

Sieving is one of the most versatile and attractive techniques for the determination of PSD of granules in dry state [49]. The average particle size of the PZB-milled extrudates was almost similar around 500 microns. For each polymer batch, a low percentage of powder below 150 μm (100# BSS sieve) and above 500 μm (30 # BSS sieve) was found. The % cumulative retention on 60 # sieve (250 µm) was observed to be 78.55%, 81.60%, 87%, and 86.77% for milled extrudates prepared using HPMC, Eudragit EPO, Affinisol 15 LV, and Kollidon VA64, respectively. Therefore, it can be concluded that fines below 250 µ were comparatively less observed in milled extrudates with Affinisol 15 LV and Kollidon VA64, while slightly higher in milled extrudates with HPMC and Eudragit EPO. Refer below to Figure 8 for Sieve analysis results of Pazopanib HCl (PZB)-milled extrudates containing HPMC, Eudragit EPO, Affinisol, and Kollidon VA64. 

Extrudates are generally susceptible to hygroscopicity; hence, larger fines might have stability concerns. In addition, further processing of the milled extrudates in tablet dosage form causes a tendency to stick to the surface of the punches and leads to a picking tendency in tablets [38] with higher fractions of particles below 250 µ. 

### 3.3. Outcomes for Optimization of Process Parameters/Variables

Based on the outcome of the optimization study for polymer-type selection, Kollidon VA64 and Affinisol 15LV were chosen for further process optimization studies. A systemic DoE design, which consisted of three factors—one categorical (polymer type) and two continuous factors (screw speed and barrel temperature). The factors and responses are summarized below in Table 4.

#### 3.3.1. Effect of Process Variables on Appearance of Extrudates

Trials no. HME-9 to HME-16 were conducted to assess the impact of process variables on the appearance of extrudates. The appearance of extrudates is summarized in Table 4. Affinisol 15LV extrudates prepared at low or high screw rpm and low or high temperature resulted in clear transparent extrudates (HME-9 to 12 trial); complete solubilization of PZB-drug in its matrix and clear transparent extrudates were observed. 

Kollidon VA64 extrudates prepared at low screw speed (100 rpm) and low barrel temperature (100/130/130/130/130 °C) resulted in less transparent extrudates (HME-13 trial) while increasing screw rpm led to clear transparent extrudates (HME-15 trial), while the high temperature combined with the low or high level of screw rpm resulted in complete solubilization of PZB-drug in its matrix and clear transparent extrudates were observed (refer to trials HME-14 and HME-16). The results were in accord with the findings of Maddineni et al., where the author revealed that processing parameters such as temperature and screw speed have the same effect on nifedipine drug solubilization [38]. 

#### 3.3.2. Effect of Process Variables on Torque of Extrudates and Statistical Evaluation

**Figure 9:**
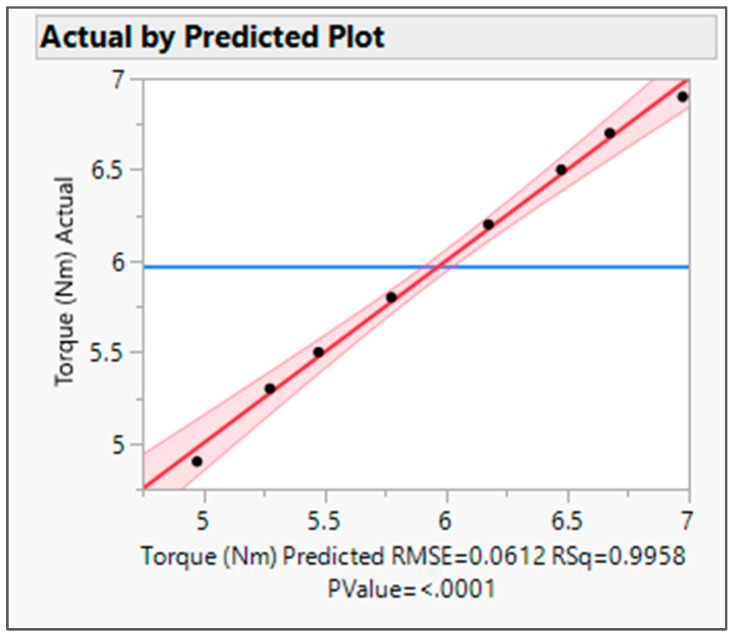
Actual by predicted plot for Torque. Blue Line-for the null hypothesis, Red Line-For Alternative Hypothesis.

The actual by the predicted plot shows the R Square value 0.9958, root mean squared error (RMSE) value 0.0612, and *p* value < 0.0001. *p* value was observed to be less than 0.05, which signifies model significance (refer to Figure 9). The RMSE value is the performance indicator of the regression model. The average difference between actual values and the predicted value by the model was found about 0.06. Hence, the model is able to predict the target value with more accuracy.

**Figure 10:**
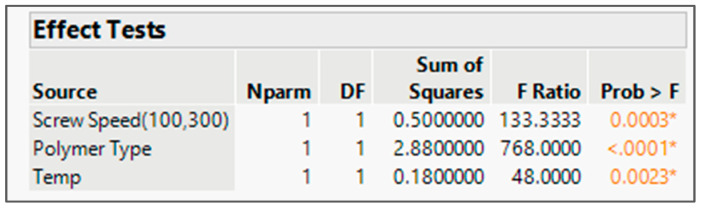
Effect Test with significance level for Torque. * and color depicts the level of significance for a factor/variable. If *p* value is less than 0.05, the * and color will appear in figure. The effect test figure indicates that the *p* value is less than 0.05 for all three input variables.

**Figure 11:**
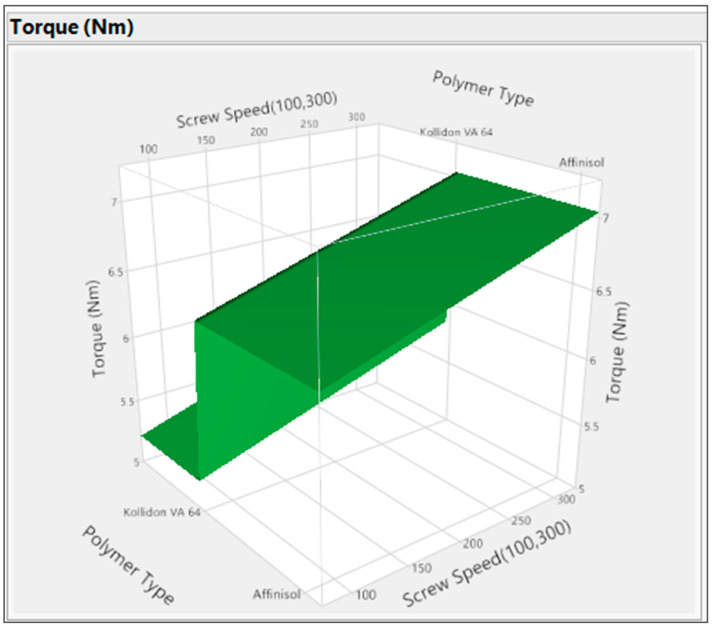
Response surface plot for response (Torque).

The effect test figure indicates that the *p* value is less than 0.05 for all three input variables (refer to Figure 10). In other words, the minimum machine torque could be obtained by choosing the Kollidon VA 64 polymer. Screw speed has a positive correlation with torque (machine processibility), which was also demonstrated in the response surface plot (Figure 11). As we increased the screw speed, excessive pressure was generated inside the extruder due to high mass load. However, barrel temperature has a negative correlation with torque (machine processibility). The polymer-type impact on the torque value was also assessed, and it was found that it has a significant impact on the torque value. Torque was found to be less with the Kollidon VA 64 polymer, while it was higher with the Affinisol polymer. This was due to the melt viscosity of these polymers.

#### 3.3.3. Effect of Process Variables on DT of Extrudates and Statistical Evaluation 

**Figure 12:**
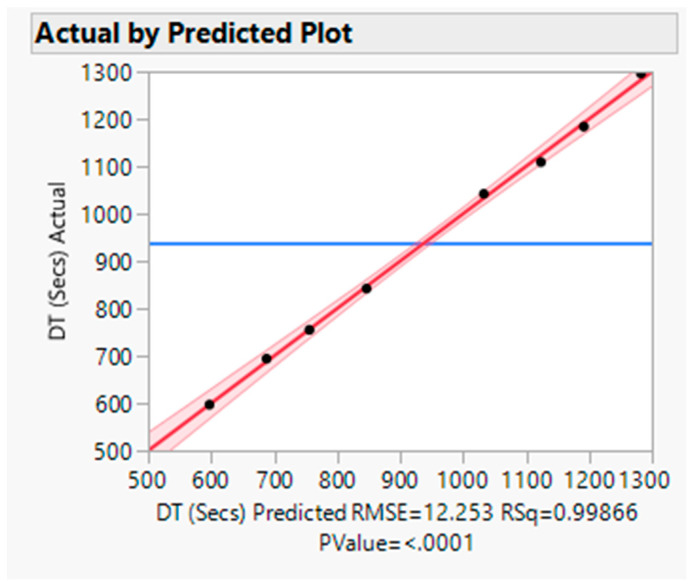
Actual by predicted plot for DT. Blue Line-for the null hypothesis, Red Line-For Alternative Hypothesis.

The actual by the predicted plot shows the R Square value 0.9987, RMSE value 12.253, and *p* value < 0.0001. Hence, the model is significant (refer to Figure 12).

**Figure 13:**
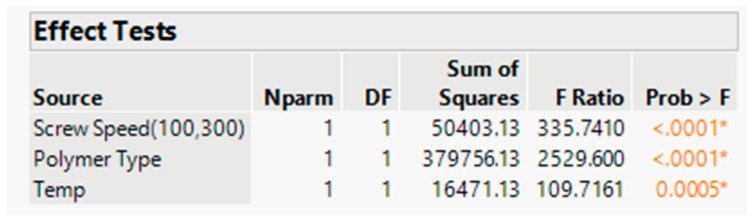
Effect Test with significance level for DT. * and color depicts the level of significance for a factor/variable. If *p* value is less than 0.05, the * and color will appear in figure. The effect test figure indicates that the *p* value is less than 0.05 for all three input variables.

**Figure 14:**
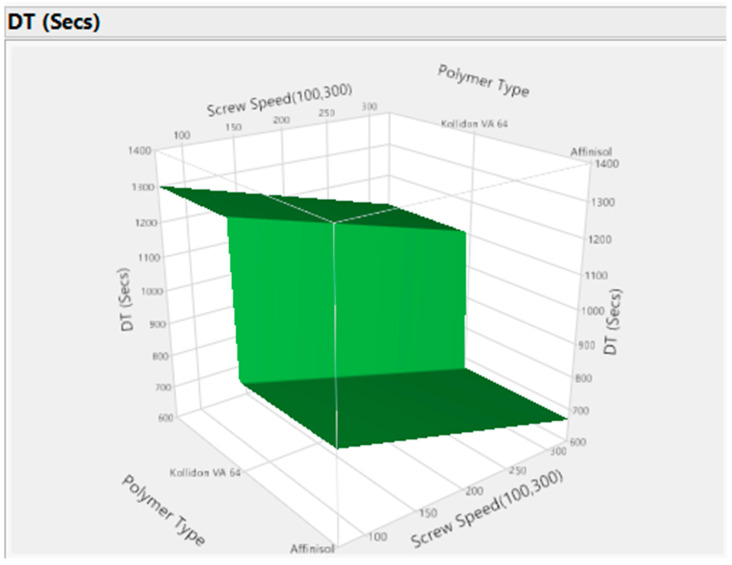
Response surface plot for response (DT).

**Figure 15:**
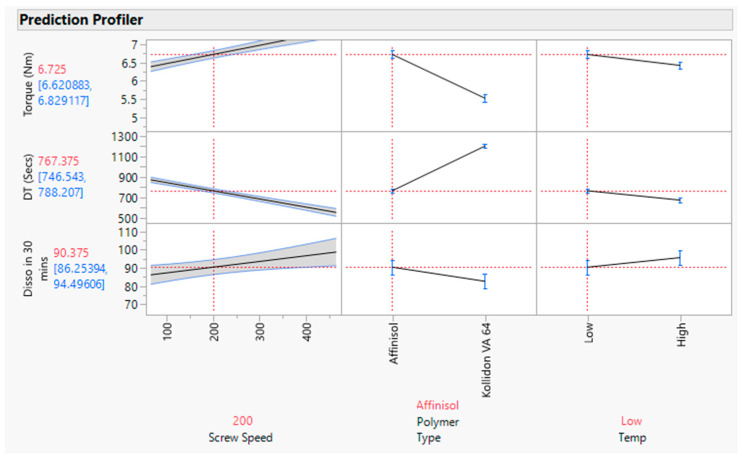
Prediction profiler for input dependent variables and output variables. Black solid line depicts the trending for studied input & its output variables.

The effect test figure indicates that the *p* value is less than 0.05 for all three input variables (refer to Figure 13). In other words, the minimum DT can be achieved with the Affinisol polymer; however, screw speed has a negative correlation with DT, which was also demonstrated in the response surface plot (Figure 14) and prediction profiler (Figure 15).

#### 3.3.4. Effect of Process Variables on Dissolution of Extrudates and Statistical Evaluation

**Figure 16:**
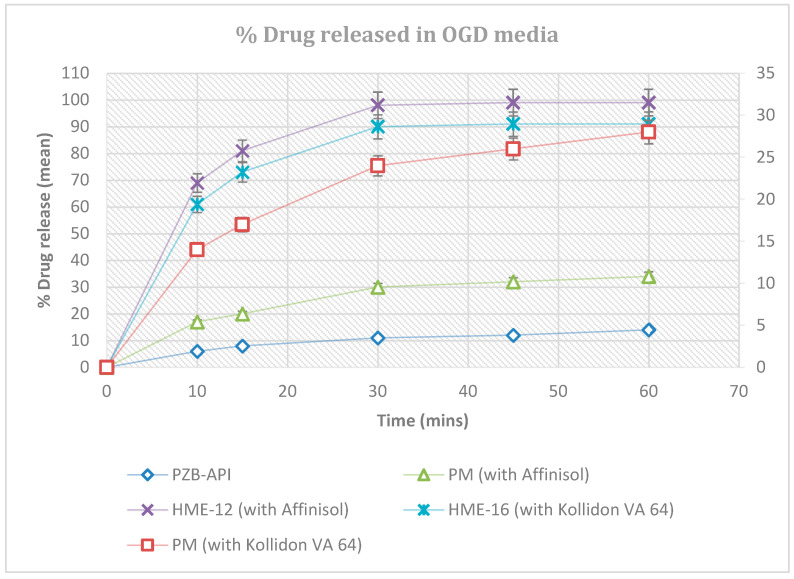
Comparative dissolution profile of Pazopanib HCl API, physical mixtures, and its extrudates.

The dissolution profiles of the drug compound PZB, its extrudates, and corresponding physical mixtures were evaluated. The comparative dissolution profile of Pazopanib HCl API, physical mixtures with Kollidon VA 64 and Affinisol polymers, and its extrudates are depicted in Figure 16. The percent drug release rate of the physical mixtures was slightly better in comparison to the pure PZB-drug substance because the water-loving polymer Affinisol can assist in wetting the extrudate particles and act to solubilize them. The drug–polymer extrudates significantly increased drug release within 30 min, and this clearly reveals a significant improvement in dissolution performance. 

Drug–polymer extrudates prepared using Kollidon VA64 and Affinisol 15LV depicted a faster and complete release, which might be due to the water-soluble vinylpyrrolidone-vinyl acetate copolymer chain. Strojewski et al. have also discussed that copovidone polymer can be used to enhance the in vitro dissolution of poorly soluble drugs [46]. HME extrudates manufactured with Kollidon VA64 and Affinisol 15LV had strong bonding between drug and polymers due to the amalgamation of PZB-drug in the melted polymeric matrix, while the physical mixture had a weak drug-to-polymer interaction. 

The physical mixture of PZB-drug with both polymers showed a slower and incomplete % drug release, which might be due to weak physical bondings such as hydrogen bonds, Vander Waals forces, or ionic interaction with the Pazopanib (PZB) drug (55, 56). Hence, the significant differences in the dissolution profile between the physical mixture and drug extrudates could be attributed to these bonding properties. 

##### Statistical Evaluation

**Figure 17:**
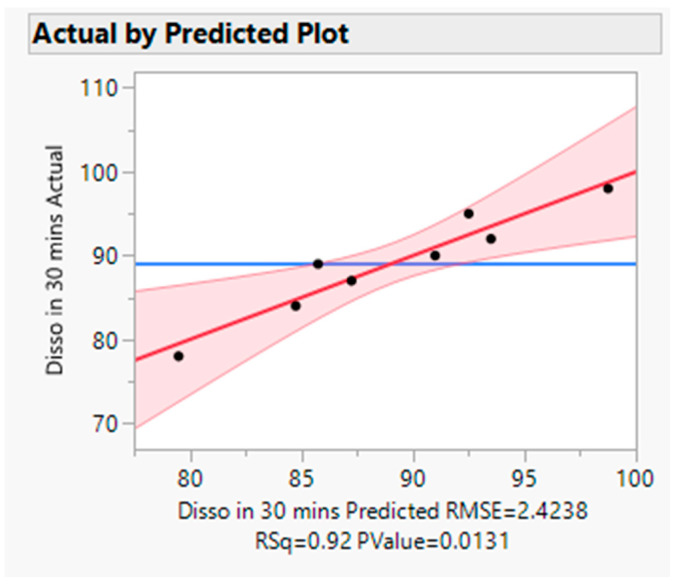
Actual by predicted plot (Dissolution). Blue Line-for the null hypothesis, Red Line-For Alternative Hypothesis. If the horizontal blue line is contained by the red region, then the whole model test is not significant at the alpha = 0.05. If the blue line is not contained within the red region, then the whole model test is significant at the same level.

**Figure 18:**
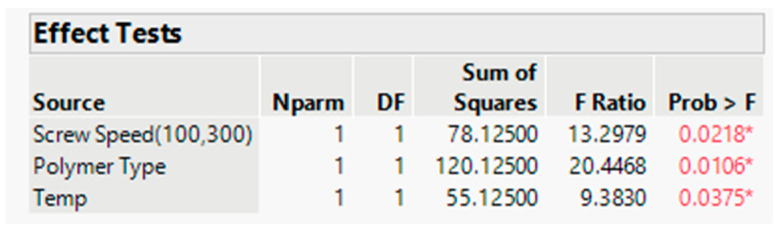
Effect Test for Dissolution (in 30 min). * and color depicts the level of significance for a factor/variable. If *p* value is less than 0.05, the * and color will appear in figure. The effect test figure indicates that the *p* value is less than 0.05 for all three input variables and dissolution data is significantly impacted by studied input variables.

**Figure 19:**
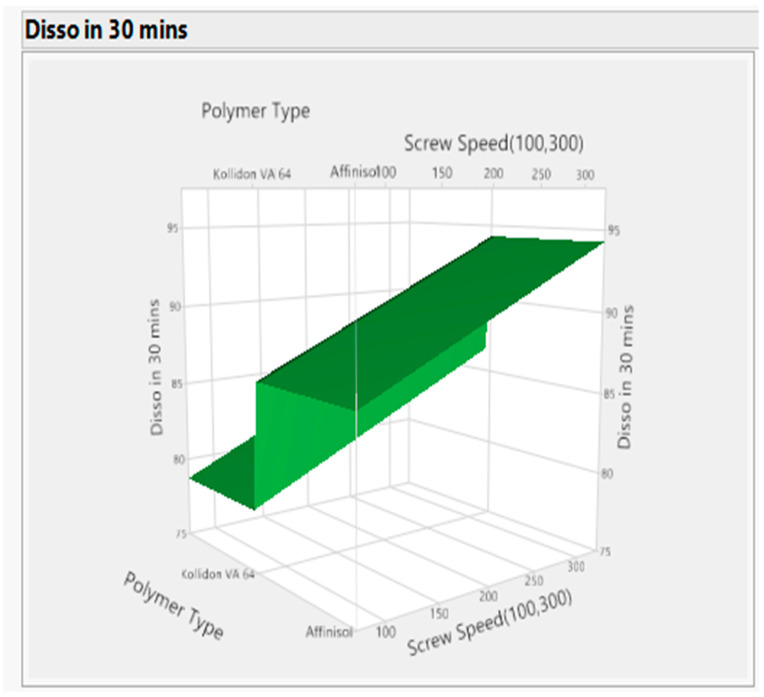
Response surface plot for response (Dissolution).

The actual by the predicted plot shows the R Square value 0.92, RMSE value 2.4238, and *p* value < 0.05, which signifies model significance (refer to Figure 17). The effect test figure indicates that the *p* value is less than 0.05 for all three input variables (refer to Figure 18). The significance order for the dissolution response is polymer-type > screw speed > barrel temp. In other words, the dissolution increased with increasing screw speed with the Affinisol polymer at low barrel temperature. This was also demonstrated in the prediction profiler (refer to Figure 15) and response surface plot (refer to Figure 19).

### 3.4. Moisture Uptake Study/Hygroscopicity

It is well-known that moisture uptake can greatly impact the performance and physical stability of manufactured extrudates (solid dispersions) by the HME technique. The hygroscopicity of polymeric materials and other excipients is well recognized to have a significant impact on the physical stability of amorphous pharmaceuticals present in solid dispersions since these materials can more readily transform into crystalline forms with increased moisture concentrations. The Affinisol 15LV polymer is less hygroscopic than the Kollidon VA 64 polymer [55]. To investigate this, the hygroscopicity of PZB-drug, polymers, and extrudates was investigated at different stability conditions. Kollidon VA64 formed lumps under ACC stability conditions while remaining in powder form at 25 ± 2 °C and 60 ± 5% relative humidity after 7 days, while the pure drug and Affinisol 15 LV polymer remained dry powders at both the stability conditions. It is surprising to note that the moisture uptake of extrudates has the same tendency as polymers, indicating that the hygroscopicity of an HME extrudate can be kept at a minimum by selecting a polymer with a lower hygroscopicity such as Affinisol 15LV. Therefore, Affinisol extrudates have less chance of changing the performance or physical stability of the extrudates. Kollidon VA 64 extrudates have 5–6% *w*/*w* moisture uptake, which is more likely to convert into crystalline form. Affinisol exhibits this anticipated change in Tg to a lesser extent as the moisture content increases much less compared to Kollidon VA64, thus minimizing the impact of water on product performance.

### 3.5. Characterization of Optimized Drug–Polymer Extrudates by HSM 

**Figure 20:**
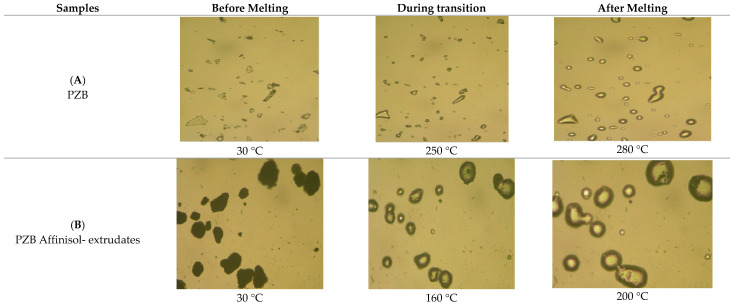
Hot-stage microscopy for Pazopanib drug and Pazopanib–polymer extrudates at 250× magnification scale.

A hot-stage microscope [ makes it simple to discriminate between crystalline and amorphous materials [. HSM can provide visual evidence about drug–polymer miscibility and its conversion to amorphous solid dispersion [56]. Images for the pure drug and drug–polymer extrudates were taken under a light microscope, and they are shown in the figure below [Figure 20]. The Pure PZB-drug began to melt at approximately 250 °C and, subsequently, completely melted at 280 °C. It was noticed that the PZB–polymer extrudates were completely melted and transformed into an amorphous state in the extrudates produced at 160 °C, which indicates that the glass transition temperature of the Pazopanib drug decreased, and molten mass was observed at the lower temperature. Hence, it can be concluded that the HME process provides adequate shear forces, which is not feasible by physical mixing only. 

### 3.6. PXRD and Polymorphic Form Stability

**Figure 21:**
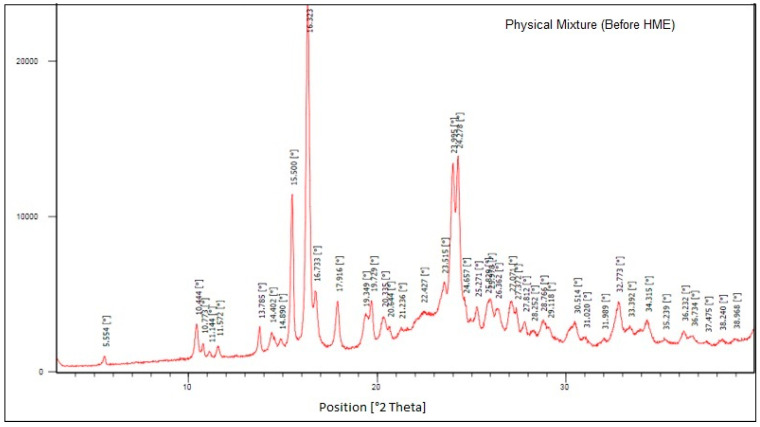
XRD of Physical mixture of Affinisol 15LV and PZB-Drug.

**Figure 22:**
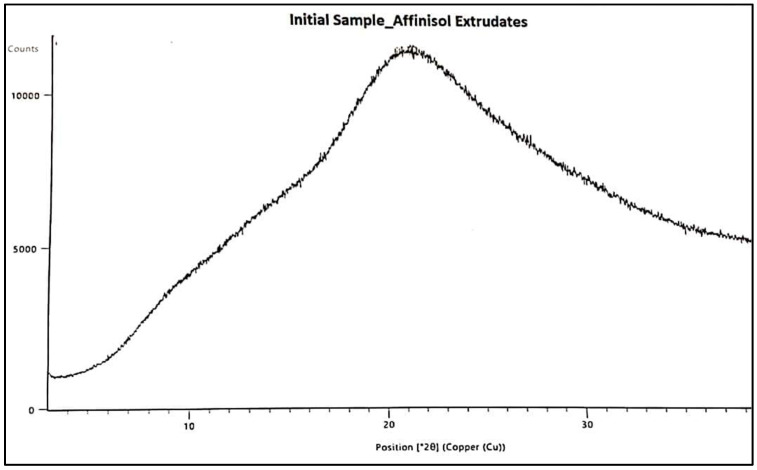
XRD of Initial sample of Affinisol PZB-Drug Extrudates.

**Figure 23:**
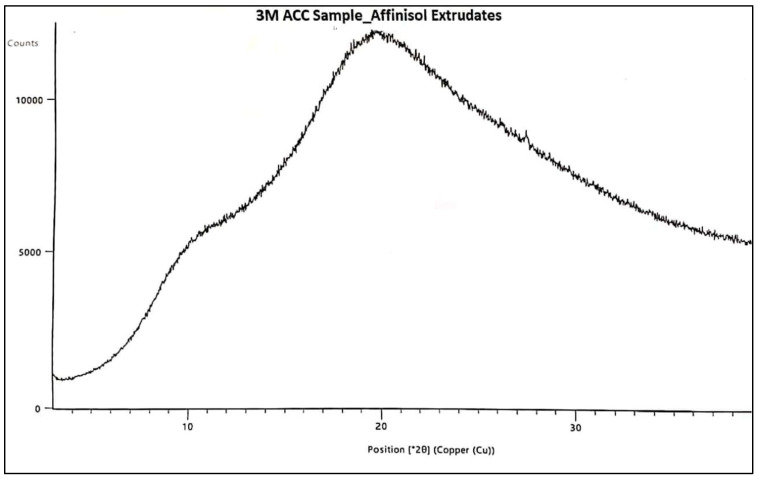
XRD of 3M ACC sample of Affinisol PZB-Drug Extrudates.

In this study, PXRD was used to assess the polymorphic form of the physical mixture, and initial and stability samples of the final prototype, i.e., Affinisol extrudate. The XRD was also carried out for crystalline drug i.e. Pazopanib hydrochloride (Refer Figure 21). The sharp and intense 2-theta peak was observed at 16.323. The PXRD patterns of initial and stability samples are presented below in Figure 22 and Figure 23, respectively. The X-ray patterns of initial and 3M ACC samples were found to be consistent, which confirms that the amorphous form of Pazopanib HCl was maintained during stability and no recrystallization occurred.

### 3.7. Stability Studies

It is known that the amorphous form of a drug compound has a faster and more complete drug release in comparison to the crystalline form. Both extrudate formulations showed an almost complete and faster drug release, whereas crystalline PZB-drug showed an incomplete drug release (refer to dissolution data in Section 3.3.4; Effect of process variables on Dissolution of extrudates and statistical evaluation). If milled extrudates show a slower and incomplete release tendency during a stability study, then this might be due to the occurrence of recrystallization. Hence, dissolution is considered an important critical quality attribute (CQA) for the stability assessment of extrudates [56]. 

The results of our stability study under ACC and long-term storage conditions up to 3 M are summarized below in Table 5. There was no significant change in the appearance, assay (drug content,) and dissolution after storage. Therefore, it can be concluded that drug extrudates are stable up to 3 M under ACC and long-term storage conditions.

### 3.8. Pharmacokinetic and Statistical Analysis

**Figure 24:**
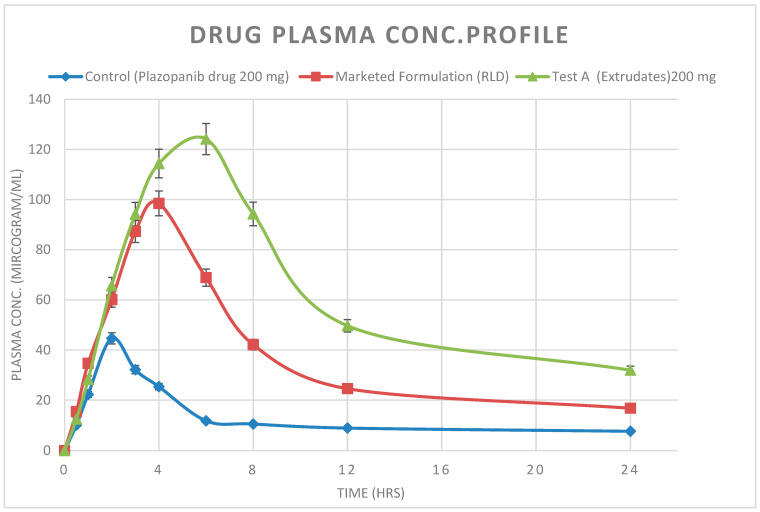
Comparative blood plasma concentration (µg/mL) at different time points for Pazopanib drug and its formulation.

Pazopanib plasma concentration was plotted against time to obtain the concentration–time profiles, which were used to determine the peak blood concentration (C_max_) and time to achieve the peak concentration (T_max_). Non-compartmental pharmacokinetic analysis was conducted to calculate the area under the curve from 0 to 24 h [(AUC)_0-24_). The values of C_max_ and T_max_ for the test preparation were obtained by actual observations. All data were presented as mean ± standard deviation. In order to determine the area under the curve from 0 to 24 h, a non-compartmental PK study was performed using Win-Nonlin PK/PD software (version 8.1). Comparative blood plasma concentration (µg/mL) at different time points for Pazopanib drug and its formulation are depicted in below Figure 24.

Pharmacokinetic parameters for PZB-drug, the marketed formulation (Votrient Tablets 200 mg), and the prepared Test prototypes, i.e., Test A (Extrudates), were calculated using Phoenix WinNonlin software and are summarized below in Table 6.

Test formulation A (PZB–Extrudates using Affinisol Polymer) has significantly higher bioavailability (AUC) in comparison to the PZB-drug substance and reference formulation (Votrient Tablets). PZB-drug has a much lower BA due to its low aqueous solubility and inconsistent oral drug absorption. Further, no lag was observed for all the studied formulations. The Test-A (PZB–Extrudates using Affinisol Polymer) formulation has a 6 h Tmax, while the reference marketed formulation has the same Tmax, i.e., 4 h. This might be due to the slightly slower absorption during the initial period, but it continued for a prolonged time. The AUC value was, in increasing order, PZB-drug < reference formulation < PZB–Extrudates.

## 4. Conclusions

In this study, PZB–Affinisol 15 LV amorphous solid dispersion (extrudates) was successfully prepared by the hot-melt extrusion technique. Hot-stage microscopy (HSM) analysis showed that PZB successfully changed from a crystalline state to an amorphous state during the HME process. The dissolution data were also consistent and showed a complete release for 3 months under accelerated and long-term stability conditions. The dissolution data also confirmed that there was no retardation of drug release due to stability issues because of the consistent amorphous state of the prepared Affinisol extrudates. The selection of the polymer/plasticizer and HME processing parameters are important concerns in the preparation of solid dispersions of poorly soluble drug compounds. A systematic experimental approach, i.e., custom DoE design and definitive screening design (JMP Software version 14.0 by SAS), was employed to evaluate the impact of the formulation and process variables on extrudates’ critical quality attributes (CQAs), respectively. The results showed that extrudates prepared using a plasticizer in comparison with those without a plasticizer make manufacturing processibility easier by improving drug–polymer miscibility. Initially, Kollidon VA64 and Affinisol 15LV polymers were chosen based on the visual appearance of the prepared extrudates. Both Kollidon VA64 and Affinisol 15LV were able to formulate clear transparent extrudates. Further, the Affinisol 15 LV polymer was selected due to its lesser moisture uptake tendency in comparison to Kollidon VA 64. Hence, PZB–Affinisol extrudates have better drug product stability during storage; hence, there is no chance of its recrystallization from the amorphous form. The *p* value was found to be below 0.05 for both the process parameters, i.e., screw speed and barrel temperature; hence, this showed a significant impact on machine torque (processability), DT, and dissolution. Extrudates prepared using a 1:2 drug-to-polymer ratio (Affinisol 15 LV) were obtained with superior in vitro dissolution and minimum disintegration time in comparison to the Kollidon VA64 extrudates. Compared with the free Pazopanib hydrochloride (PZB) drug and commercially marketed product (Votrient^®^Tablets), the bioavailability (AUC) of PZB–Extrudates using Affinisol Polymer (Test formulation A) was improved by 4.79- and 1.66-fold, respectively. Based on these results, Affinisol 15LV could serve as an efficient polymeric carrier to prepare a solid dispersion containing a BCS Class II drug via the hot-melt extrusion process.

The bioavailability (AUC) and pharmacokinetic parameters were evaluated on animals on different formulations and, hence, further BA/BE studies need to be carried out on human volunteers. Currently, the presence of nitrosamine impurities in drug products, which may cause cancer in patients, is a major concern in the pharmaceutical/healthcare industry. Drug product components must be assessed for their likelihood of forming nitrosamine impurities. Thus, a nitrosamine risk assessment along with a control strategy must be ensured for the final optimized formulation. 

In the future, Affinisol extrudates will be converted into a solid unit dosage form, and further stability studies should be conducted to assess this form in different primary packs as per different ICH stability zones. 

## Figures and Tables

**Table 1:** Prototype formula composition of extrudates.

S. No.	Ingredients	Function	Qty. (mg/Unit)
1.	Pazopanib HCl	API	200,000
2.	Kollidon VA64/HPMC/ Eudragit EPO/Affinisol 15LV	Polymer	400,000
3.	Poloxamer 188	Plasticizer	40,000

**Table 2:** DoE matrix for formulation and process variables.

Input (Independent) Variables		Levels			
A. Formulation variables					
i	Type of Polymer	Kollidon VA64	HPMC	Eudragit EPO	Affinisol 15LV
ii	Plasticizer (Poloxamer 188)	Yes (10%)	No (0%)		
B. Process variables					
i	Type of Polymer	Kollidon VA64	Affinisol 15LV		
ii	Screw RPM	100	300		
iii	Barrel Temperature	Low	High		
Output (Dependent) variables		Target range			
Y1	Extrudates Appearance	Yes or No			
Y2	Torque	Minimum			
Y3	Disintegration Time of extrudates	Minimum			
Y4	Dissolution (30 min) ^#^	Maximum			

^#^ Response studied for Process DoE.

**Table 3:** Factors and responses for optimization of formulation variables.

Trial No.	Polymer	Plasticizer	Plasticizer Level (% *w*/*w*)	API	Torque (Nm)	Appearance	DT
HME-1	HPMC	Poloxamer 188	10	PZB	7.4	Brownish in color	15 min 32 s
HME-2	Eudragit EPO	Poloxamer 188	10	PZB	8.7	White and opaque	23 min 06 s
HME-3	Affinisol 15LV	Poloxamer 188	10	PZB	5.7	Clear and transparent	11 min 45 s
HME-4	Kollidon VA64	Poloxamer 188	10	PZB	4.9	Clear and transparent	18 min 11 s
HME-5	HPMC	-	0	PZB	7.9	Brownish in color	19 min 18 s
HME-6	Eudragit EPO	-	0	PZB	9.8	White and opaque	27 min 16 s
HME-7	Affinisol 15LV	-	0	PZB	6.8	Clear and transparent	13 min 46 s
HME-8	Kollidon VA64	-	0	PZB	5.6	Clear and transparent	22 min 54 s

**Table 4:** Optimization of process parameters with input and output variables.

Trial No.	Input Variables			Output Variables/Responses				
	Polymer	Screw Speed	Barrel Temperature	Torque (Nm)	Appearance	DT	Dissolution in 30 min	% Moisture Uptake
HME-9	Affinisol 15LV	100	100/130/130/130/130 (Low)	6.5	Clear transparent	14 min 02 s (842 s)	87	0.54
HME-10	Affinisol 15LV	100	100/180/180/180/180 (High)	6.2	Clear transparent	12 min 35 s (755 s)	95	0.91
HME-11	Affinisol 15LV	300	100/130/130/130/130 (Low)	6.9	Clear transparent	11 min 34 s (694 s)	92	0.61
HME-12	Affinisol 15LV	300	100/180/180/180/180 (High)	6.7	Clear transparent	09 min 57 s (597 s)	98	0.98
HME-13	Kollidon VA64	100	100/130/130/130/130 (Low)	5.3	Less transparent	21 min 36 s (1296 s)	78	5.11
HME-14	Kollidon VA64	100	100/180/180/180/180 (High)	4.9	Clear transparent	19 min 44 s (1184 s)	84	6.25
HME-15	Kollidon VA64	300	100/130/130/130/130 (Low)	5.8	Clear transparent	18 min 29 s (1109 s)	89	5.58
HME-16	Kollidon VA64	300	100/180/180/180/180 (High)	5.5	Clear transparent	17 min 22 s (1042 s)	90	6.49

**Table 5:** Stability data of Affinisol Extrudates in ACC and Long-Term Storage Conditions.

Sample	Stability Condition	Period	Appearance	Assay	Dissolution in 30 min
Affinisol Extrudates	40 ± 2 °C and 75 ± 5% relative humidity (ACC)	Initial	Free-flowing powder	98.7	99
		1 M	Free-flowing powder	98.1	98
		3 M	Free-flowing powder	98.2	97
	25 ± 2 °C and 60 ± 5% relative humidity (Long-term storage)	Initial	Free-flowing powder	98.7	99
		1 M	Free-flowing powder	98.4	97
		3 M	Free-flowing powder	98.0	96

**Table 6:** Pharmacokinetic statistical parameters for different formulation prototypes.

Formulations	Tmax (h)	Cmax (µg/mL)	C Last (µg/mL)	AUC Last (µg.h/mL)	T lag (h)
PZB-Drug/API	2	44.7	7.6	308.675	0
Reference/ (Marketed Formulation)	4	98.5	16.8	890.875	0
Test A-Extrudates	6	124.2	32	1479.875	0

## Data Availability

The data presented in this study are available on request from the corresponding author. All data is provided in the manuscript.
